# Perceived stress and humanistic care ability among Chinese healthcare workers: The chain mediating role of social support and life satisfaction

**DOI:** 10.3389/fpsyg.2022.1029265

**Published:** 2022-11-09

**Authors:** Zonghua Wang, Langlang Xie, Zeping Liang, Jiangshan Fan, Liqi Fan, Jing Deng, Xia Xu

**Affiliations:** ^1^Department of Clinical Nursing, School of Nursing, Army Medical University, Chongqing, China; ^2^Department of Health Management and Geriatric Nursing, Daping Hospital, Chongqing, China; ^3^Department of Nursing, Daping Hospital, Chongqing, China; ^4^Department of Respiratory and Critical Care Medicine, Southwest Hospital, Chongqing, China

**Keywords:** perceived stress, humanistic care ability, social support, life satisfaction, clinical nursing teachers

## Abstract

Previous studies have indicated high perceived stress and its relationship with life satisfaction among healthcare workers. However, most of the existing studies have focused on the investigation and evaluation of the humanistic care abilities among nurses, but few studies revealed the levels of humanistic care ability among other healthcare workers including doctors and technicians. The study aimed to investigate the perceived stress and humanistic care abilities among Chinese healthcare workers. In addition, we further examined the mediating and moderating effects of social support and life satisfaction. A convenience sample of 955 health professionals from 29 hospitals in China was recruited to fill out the questionnaires about perceived stress, humanistic care ability, social support, and life satisfaction. The correlation and multivariate logistic regression analysis were carried out by SPSS 24.0. The Hayes SPSS macro program process (version 2.16.3) was used to analyze the significance of mediating and moderating model. The findings indicated that humanistic care ability was negatively associated with perceived stress and positively correlated with social support and life satisfaction. The effect of the path “perceived stress → social support → humanistic care ability” was −0.017, and the path “perceived stress → life satisfaction → social support → humanistic care ability” was −0.129. The current study contributed to a better understanding of humanistic care abilities and influential factors in Chinese healthcare workers. Thus, it may suggest studies on interventions to interventions to alleviate or eliminate the negative impact of perceived stress and improve humanistic care abilities.

## Introduction

Humanistic care (Silva, 2013) is patient-centered care that emphasizes that each patient is different and needs to be treated with respect while responding to their unique needs and preferences depending on their current situation. This means (Zhang et al., 2021) that the provision of care must be respectful of and responsive to each patient's needs and values and that the patient should have an opportunity to provide their opinion with respect to the clinical decisions that affect them. This type of care takes into account that each patient feels, perceives, and responds to the various situations they encounter differently. According to humanistic nursing theory (O'Conoor, 1993), humanistic care is characterized by the interactions between healthcare workers and patients to promote quality of life. Humanistic care promotes “a positive and trusting relationship between the caregiver and a patient, characterized by collaboration, dignity, empathy, and trust.” (Zhang et al., 2021). Without the quality of humanism, healthcare workers may lack the attributes of integrity, honesty, and compassion which are essential in the healthcare profession (Mustika and Soemantri, 2020), especially for those who work in the areas of hospice care, palliative care (Wu and Volker, 2012), chronic disease care (Gater et al., 2015), and cancer care (Gao et al., 2021b). A clinical practice (França et al., 2013) lacking humanism may prevent effective communication between healthcare workers and patients, may cause the relationship between staff and patients to deteriorate, may result in a loss of trust between healthcare workers and patients, and may lead to an increase in conflict (Zhong et al., 2021) between staff and patients. This will worsen patients' clinical outcomes and reduce their overall quality of life.

The “Healthy China 2030” Plan was developed by the Chinese government as a national strategy to improve the health of the general population. This includes the improvement of healthcare workers' humanistic care abilities to strengthen humanistic care in health services and promote a harmonious relationship between patients and medical staff (Tan et al., 2019). Humanistic healthcare abilities refer to a healthcare worker's capability to identify the needs and preferences of the patient, understand how the patient is feeling in their current circumstances, and employ inner characteristics of humanity, morality, and knowledge in clinical practice. The ultimate aim of humanistic care is to promote better health outcomes and improve the patient's overall quality of life (Watson, 2007). Shiau and Chen (2008) note that health professionals need to be reflective, critical, and flexible to provide satisfactory humanistic healthcare.

If we consider healthcare workers' perspectives, we find that emotional disorders, such as anxiety and depression, are generally associated with perceived stress. Stress has also been linked with cognitive dysfunction in medical staff, such as impaired memory, compassion fatigue, and burnout syndromes, which eventually jeopardizes their job satisfaction (Gao et al., 2021a) and quality of life (Shiau and Chen, 2008; Deng et al., 2019a; Wang et al., 2020). From an organizational perspective, high levels of work-related stress are positively associated with increased sick leave due to psychiatric illness (Deng et al., 2019a) and increased intention to resign (Xu et al., 2021), and therefore can lead to staff shortages in clinical settings (She et al., 2021). Stress may also reduce productivity and increase the incidence of errors. If we consider the connection between stress and care quality, we see that healthcare workers who report high levels of stress, fatigue, and burnout pay less attention to patient preferences, needs, and values and may be unable to carry out “patient-centered care” with more humanism (Zhang et al., 2021).

Certain studies (Yang et al., 2018; Fisher et al., 2022) have demonstrated that social support was a mediating factor in the negative relationship between perceived stress and life satisfaction. In a previous study we conducted, we found a negative correlation between perceived stress and life satisfaction (*r*= −0.498) (Tough et al., 2017). Our results showed that individuals with extremely high levels of perceived stress rarely felt satisfied with themselves and were likely to receive lower social support. Harmonious interpersonal relationships have been considered to be one of the standards of good psychological health (Santini et al., 2015). An increasing number of studies have indicated that poor social interactions negatively impact mental health (Siaw and Lee, 2019). However, it remains unclear what role social support and life satisfaction play in the connection between perceived stress and humanistic care ability. With high levels of social support and life satisfaction, healthcare workers were expected to mainly manifest in their interaction with colleagues and patients. This was assumed to increase humanistic care awareness and behavior.

Most of the existing studies have focused on an investigation and evaluation of the humanistic care abilities of nurses (Gao et al., 2021a) and nursing students (Shiau and Chen, 2008), but few studies have examined the humanistic care abilities of various medical workers, including doctors, nurses, and technicians. Since studies have shown that healthcare disciplines are interrelated and interdependent, there has been an increasing need for interdisciplinary cooperation across health professionals (Siaw and Lee, 2019). Therefore, this study aimed to investigate the humanistic care abilities of different types of Chinese medical workers. In addition, we further aimed to examine the relationship between as well as the mediating and moderating effects of humanistic care ability and perceived stress, social support, and life satisfaction. We expect that our results will add to the body of knowledge already available by encouraging a better understanding of the humanistic care abilities of and influential factors on Chinese healthcare workers. Our results may also assist in the development of targeted training programs to improve the humanistic care abilities of medical staff in clinical settings.

## Materials and methods

### Participants

This cross-sectional survey was conducted in January 2021. The study adopted a convenience sampling approach and recruited a total of 955 health professionals from 29 hospitals in China. The advertisement for the survey was posted on bulletin boards across clinical departments, so any potential participant who met the inclusion criteria could fill out the survey by a paper copy or through an electronic link. The inclusion criteria were: the participants had (1) to be on active duty as a doctor or a nurse or a medical technician; and (2) to give informed consent and participate voluntarily. Participants were excluded if they: (1) were not involved in clinical work for more than half a year; (2) were informal employees, such as intern students, visiting scholars, and so forth; and (3) were medical personnel working in communities or primary care outpatients.

### Measures

The general information and humanistic care ability, perceived stress, social support, and life satisfaction scores were collected by self-designed and validated scales. The general information included demographic and working data of gender, age, education level, marital status, professional title, job position, hospital grade, working department, and years.

#### Humanistic care ability

The scale was developed by one of our authors, Deng (Deng et al., 2019a), in 2016 using the Delphi method through three rounds of consultation with experts. The scale comprised a total of 42 items which were divided into four dimensions, including “respecting the independence and initiative of patients' personality” (8 items), “satisfying patients' reasonable medical requirements” (11 items), “satisfying patients' physiological, psychological, and social needs” (12 items), and “implementing humanistic care” (11 items). Each item was rated on a 5-point Likert scale, with 5 representing the best practice and 1 representing the poorest practice in humanistic care. A higher score meant better humanistic care ability. The scale was extensively validated and used, with an overall Cronbach's α coefficient of 0.93 and the dimensional Cronbach's α ranging from 0.930 to 0.950 (Deng et al., 2019a). The overall Cronbach's α in our study sample was 0.984, indicating good internal consistency.

#### Perceived stress

This study adopted the Chinese perceived stress scale (CPSS) to measure perceived stress over the month preceding the study. This 14-item scale consists of two dimensions, with seven items related to “sense of control” and seven items related to “sense of tension.” Each item was scored on a 5-point Likert scale. The higher the participant's total score, the higher the level of their perceived stress. The total score ranged from 0 to 56. The Cronbach's α coefficient was from 0.77 to 0.86 for the total scale and the two subscales (Xu et al., 2021). If a participant had a total CPSS score of more than 26, then this was recognized as health risk stress (HRS) (Lu et al., 2019). The Cronbach's α was 0.756.

#### Social support

This study employed the social support rating scale (SSRS) (Tomás et al., 2016) designed and compiled by Xiao et al. This scale included three dimensions: subjective support, objective support, and support utilization. The scale comprised a total number of 10 items and total scores ranged between 12 and 66. A higher total score indicated better social support. In this study, the Cronbach's α coefficient of the scale was 0.813.

#### Life satisfaction

The Chinese version of the satisfaction with life scale (SWLS) was originally developed by Diener et al. (1985) showing a satisfactory Cronbach's α coefficient of 0.84 (Yang et al., 2018). We utilized this scale to measure the participant's satisfaction with life and subjective well-being (SWB). This scale consisted of five items and was rated on a 7-point Likert scale, with 1 indicating “totally disagree” and 7 indicating “totally agree.” The higher the total score was, the greater the participant's life satisfaction was said to be. In our study, the Cronbach's α-value was 0.927.

### Data collection

The survey was self-administered anonymously using an online platform. The online questionnaire was designed with two parts: an information sheet and a scale sheet. The information sheet provided a description of the study, instructions on how to complete the scales, and an informed consent form. After providing informed consent, the participants were presented with the assessment sheet. We requested a group of healthcare administrators to assist with the distribution of the survey. An online training presentation was organized for the study participants to clarify the purpose of the study and guidelines were provided to ensure better data collection. A total of 955 questionnaires were collected for analysis.

### Statistical analysis

The SPSS 24.0 and R software were used for statistical analysis. When carried out the Kolmogorov–Smirnov test, our data did not show a normal distribution. Descriptive statistics were summarized by percentage, number, median, and interquartile range. The Mann–Whitney U-test and Kruskal–Wallis H rank-sum test were used to compare the scores of humanistic care ability, perceived stress, social support, and life satisfaction with different demographic characteristics. A Spearman correlation analysis was conducted between various variables using R software (version 4.2.1, released on 2022-06-23). According to the extreme grouping method of psychometrics (Kusier and Folker, 2021), the participants were divided into three groups by humanistic care ability scores. The first 27% of the participants who had high levels of humanistic care ability were called the high group, the last 27% were called the low group, and the middle 46% were called the medium group. Then, multivariate logistic regression was used to analyze the influence factors of humanistic care ability. The Hayes SPSS macro program process (version 2.16.3) was used to analyze the significance of mediating and moderating model. First, we tested the moderating effect of social support and life satisfaction between perceived stress and humanistic care ability by utilizing a Model 2 process. Thereafter, we tested the chain mediating effect of life satisfaction and social support between perceived stress and humanistic care ability using the Model 6 process.

## Results

### Sample characteristics

As is shown in Table 1, our study sample included 100 male participants and 855 female participants, with a median age of 31 years [27.0, 36.0]. In respect of educational background, over two-thirds of the participants (69.6%, 665/955) held a bachelor's degree or higher, and the remaining participants had a college diploma. With regard to the professional title held by the participants, approximately two-thirds (64.4%, 615/955) held a primary title, 28.7% (274/955) held an intermediate professional title, and 6.9% (66/955) held an advanced professional title. Approximately 13.4% of the participants (123) worked at a primary hospital, 234 (24.5%) worked at a secondary hospital, and 598 (62.6%) worked at a tertiary hospital. The majority of the participants were nurses, accounting for 83.35%, and the remaining participants were either doctors or medical technicians. In respect of the number of years the participants had been working, approximately one-fifth of the participants (266/955) had been working at the same hospital for < 5 years, 34.0% (325/955) had been working for between 6 and 10 years, 26.2% (250/955) had been working for between 11 and 20 years, and 11.9% (114/955) had been working for more than 20 years. Only one-third of the participants (33.0%, 315/955) had ever reported receiving training on humanistic care.

**Table 1:** Comparisons on all scales' scores by different demographic features.

**Items**	***N***	**HCA**	**PS**	**SS**	**LS**
**Gender**
Male	100	160.0 (128.3, 190.8)	26.0 (20.0, 28.0)	42.0 (36.0, 47.8)	25.5 (20.0, 30.0)
Female	855	160.0 (128.0, 181.0)	27.0 (21.0, 29.0)	43.0 (36.0, 48.0)	25.0 (20.0, 29.0)
*Z*-value		−0.554	−1.016	−0.695	−0.827
*P*-value		0.580	0.310	0.487	0.408
**Age**					
≤ 30	463	160.0 (126.0, 183.0)	27.0 (22.0, 29.0)^abc^	40.0 (34.0, 46.0)^a^	24.0 (19.0, 29.0)^abc^
31–35	234	160.0 (130.8, 181.0)	27.0 (22.0, 29.0)^abc^	44.0 (40.0, 49.0)^bcd^	25.0 (20.0, 30.0)^abc^
36–40	150	157.0 (129.8, 174.8)	26.0 (21.0, 28.0)^abc^	44.0 (38.0, 50.0)^bcd^	26.0 (20.0, 29.0)^abc^
≥41	106	166.0 (136.3, 193.0)	23.0 (17.0, 27.3)^d^	47.0 (40.0, 51.3)^bcd^	27.0 (23.0, 30.0)^d^
*H*-value		5.353	27.180	65.972	24.509
*P*-value		0.148	< 0.001	< 0.001	< 0.001
**Education**					
College diploma	290	159.5 (127.0, 186.0)	27.5 (22.0, 29.0)^ab^	42.0 (36.0, 48.0)	24.0 (19.0, 30.0)
Bachelor degree	601	160.0 (126.5, 179.0)	27.0 (21.0, 28.0)^ab^	43.0 (36.0, 48.0)	25.0 (20.0, 29.0)
Master degree and above	64	160.0 (148.5, 181.0)	23.0 (17.0, 28.0)^c^	44.0 (36.0, 48.8)	26.5 (20.0, 30.0)
*H*-value		2.323	20.262	1.372	4.582
*P*-value		0.313	< 0.001	0.504	0.101
**Professional title**
Primary	615	160.0 (129.0, 18.04)	27.0 (21.0, 29.0)^ab^	42.0 (35.0, 47.0)^a^	24.0 (19.0, 29.0)^ab^
Intermediate	274	160.0 (127.8, 177.3)	26.0 (21.0, 28.3)^ab^	44.0 (38.0, 49.0)^b^	25.0 (20.0, 29.0)^ab^
Advanced	66	163.5 (127.5, 187.8)	23.0 (16.0, 27.0)^c^	48.0 (43.8, 52.3)^c^	28.0 (24.0, 30.0)^c^
*H*-value		0.632	21.133	45.602	22.011
*P*-value		0.729	< 0.001	< 0.001	< 0.001
**Hospital source**
Primary	123	145.0 (126.0, 164.0)^a^	28.0 (24.0, 29.0)^ab^	42.0 (35.0, 48.0)	22.0 (18.0, 27.0)^a^
Secondary	234	160.0 (131.0, 190.5)^bc^	27.0 (22.0, 29.0)^ab^	43.0 (37.0, 48.0)	25.0 (20.0, 30.0)^bc^
Tertiary	598	160.0 (128.0, 180.3)^bc^	26.0 (20.0, 28.0)^c^	43.0 (36.0, 48.0)	25.0 (20.0, 29.0)^bc^
*H*-value		11.247	14.533	1.445	11.654
*P*-value		0.004	0.001	0.485	0.003
**Job type**					
Doctor	102	161.5 (139.0, 190.0)^a^	24.0 (17.0, 28.0)^a^	44.0 (36.7, 50.0)	26.0 (20.0, 30.0)
Nurse	796	160.0 (127.0, 182.0)^abc^	27.0 (21.0, 29.0)^b^	43.0 (36.0, 48.0)	25.0 (20.0, 29.0)
Medical technician	57	150.0 (126.5, 160.0)^c^	26.0 (21.0, 28.0)^abc^	41.0 (35.0, 47.0)	24.0 (19.5, 29.5)
*H*-value		7.409	11.258	3.931	3.180
*P*-value		0.025	0.004	0.140	0.204
**Length of working**
≤ 5	266	159.0 (128.8, 182.3)	27.0 (20.0, 29.0)^abc^	39.0 (33.0, 46.0)^a^	24.0 (19.0, 29.0)^abc^
6–10	325	160.0 (130.0, 182.0)	27.0 (22.0, 28.0)^abc^	42.0 (37.0, 48.0)^bc^	25.0 (20.0, 29.0)^abc^
11–20	250	158.8 (126.0, 181.3)	27.0 (22.0, 29.0)^abc^	44.0 (38.0, 49.0)^bcd^	25.0 (20.0, 29.0)^abc^
>20	114	164.5 (128.0, 181.5)	24.0 (18.0, 28.0)^d^	46.0 (39.8, 50.0)^cd^	27.0 (22.8, 30.0)^d^
*H*-value		1.288	15.883	47.180	17.624
*P*-value		0.732	0.001	< 0.001	0.001
**Experience of training**
No	640	159.5 (131.0, 177.8)	27.0 (21.0, 29.0)	42.0 (36.0, 47.0)	24.0 (19.0, 29.0)
Yes	315	160.0 (126.0, 192.0)	27.0 (21.0, 29.0)	45.0 (38.0, 50.0)	26.0 (21.0, 30.0)
*Z*-value		−0.974	−0.388	−4.214	−3.342
*P*-value		0.330	0.698	< 0.001	0.001

N, number; HCA, humanistic care ability; PS, perceived stress; SS, social support; LS, life satisfaction.

### Humanistic care ability

The median score for the overall humanistic care ability scale in our study sample was 160.0 [128.0, 182.0]. The median score of each dimension was as follows: respecting the independence and initiative of patients' personality 31.0 [24.0, 35.0]; satisfying patients' reasonable medical requirements 42.0 [33.0, 48.0]; satisfying patients' physiological, psychological, and social needs 44.0 [36.0, 52.0]; and implementing humanistic care 42.0 [33.0, 48.0]. There was no significant difference in the overall score in participants with different demographic features and working characteristics (*P* > 0.05), except for the job type and hospital source (shown in Table 1). The scores of doctors were higher than that of medical technicians (*P* = 0.019) in this category.

### Descriptive analysis

For perceived stress, the median score among our subjects was 27.0 [21.0, 29.0] and the median score of each dimension was as follows: sense of tension 13.0 [9.0, 14.0] and sense of control 14.0 [11.0, 15.0]. In our study, over half of the participants (56.3%, 538/955) showed a CPSS score >26, indicating a high risk of stress impacting negatively their health. In respect of social support, the overall median score was 43.0 [36.0, 48.0]. The dimension of subjective support was 25.0 [21.0, 28.0], the dimension of objective support was 10.0 [8.0, 12.0], and the dimension of support utilization was 8.0 [7.0, 9.0]. Taking the scores for life satisfaction into account, we saw that the SWLS scores ranged from 5.0 to 35.0, with an overall median of 25.0 [20.0, 29.0].

### Correlations between various variables

**Figure 1:**
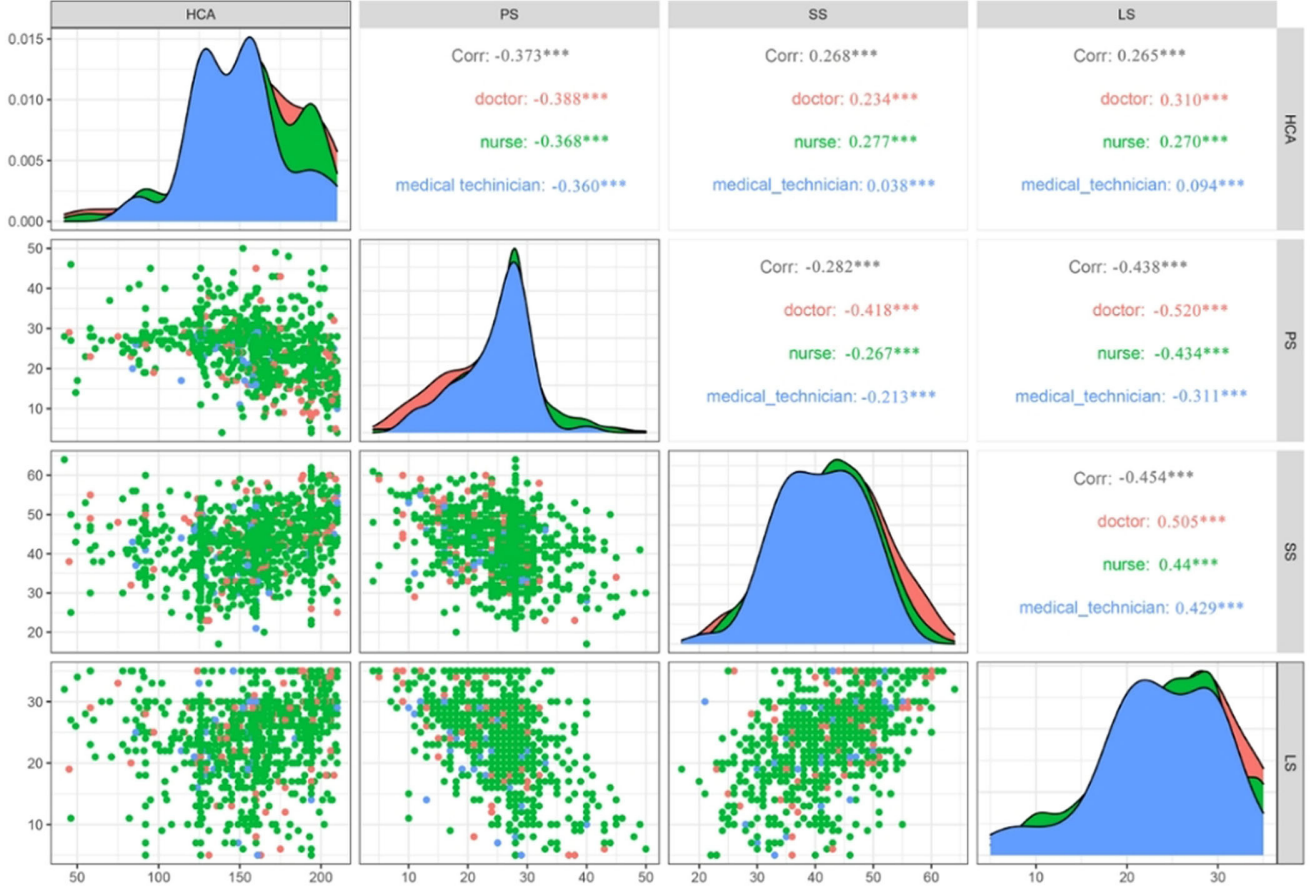
The Spearman correlations of humanistic care ability with other major variables (*n* = 955).

As is presented in Figure 1, our results show that the humanistic care ability negatively correlated with perceived stress *(r* = −0.373^***^) and positively correlated with social support *(r* = 0.268^***^) and life satisfaction *(r* = 0.265^***^). For a participant who works on different types of jobs, the coefficients between each scale maintain the same trend, which showed that doctors, nurses, and medical technicians' coefficients were 0.505 *(doctor, P*<*0.001)*, 0.440 *(nurse, P*<*0.001)*, and 0.429 *(medical technician, P*<*0.001)*, respectively.

### Multivariate logistic regression analysis

As is indicated in Table 2, if we make use of the low humanistic care ability group as the reference category, the results of multivariate logistic regressions revealed that perceived stress was a risk factor affecting humanistic care ability both in the middle-level humanistic care ability group *[B* = −*0.069, SE* = *0.015, Wald* = *22.572, P*<*0.001, OR (95.0%CI)* = *0.933 (0.907–0.960)]* and the high-level humanistic care ability group *[B* = −*0.122, SE* = *0.017, Wald* = *51.495, P*<*0.001, OR (95.0%CI)* = *0.885 (0.856–0.915)]*. For social support, the regression effect was not significant *(P* = *0.09* > *0.05)* in the middle-level group, but was significant in the high-level group *[B* = *0.057, SE* = *0.013, Wald* = *17.728, P* = *0.000, 1.058, OR (95.0%CI)* = *(1.031–1.087)]*. For life satisfaction, both the middle- and high-level groups' regression were not significant *(P* = *0.098, P* = *0.142)*.

**Table 2:** The multivariate logistic regression analysis of the factors affecting humanistic care ability.

**Group**	**Factors**	***B***	***SE***	**Wald**	***P***	**OR (95.0%CI) value**
Group with high level of humanistic care ability*	Intercept	0.019	0.827	0.001	0.982	
	Perceived stress	−0.122	0.017	51.495	0.000	0.885 (0.856–0.915)
	Social support	0.057	0.013	17.728	0.000	1.058 (1.031–1.087)
	Life satisfaction	0.026	0.017	2.160	0.142	1.026 (0.991–1.062)
Group with middle level of humanistic care ability*	Intercept	2.129	0.694	9.401	0.002	
	Perceived stress	−0.069	0.015	22.572	0.000	0.933 (0.907–0.960)
	Social support	0.019	0.011	2.868	0.090	1.019 (0.997–1.041)
	Life satisfaction	−0.023	0.014	2.744	0.098	0.977 (0.951–1.004)

*Compared to the group with low level of humanistic care ability.

### Mediating effect analysis

**Figure 2:**
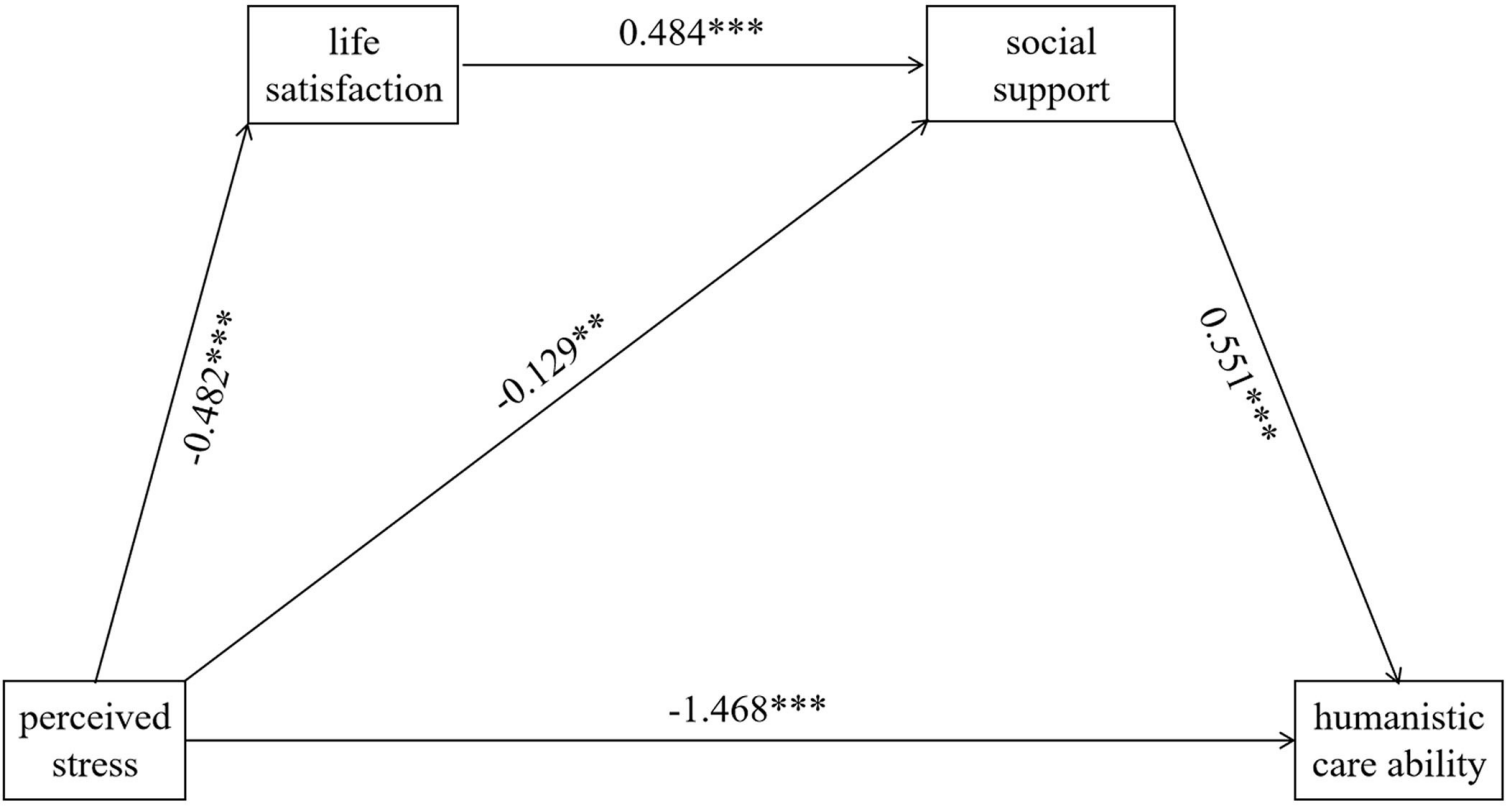
The chain mediating effect of social support and life satisfaction between perceived stress and humanistic. The ** means *P* < 0.01; the *** means *P* < 0.001.

With the SPSS macro PROCESS software, the mediating effect was conducted using Model 6 to investigate life satisfaction and social support between perceived stress and humanistic care ability. The results showed that perceived stress was a negative predictor of life satisfaction *(*β = −*0.482, P*<*0.001)* and social support *(*β = −*0.129, P*<*0.01)*. When perceived stress, social support, and life satisfaction were put into the regression model, the results indicated that perceived stress was a negative predictor of humanistic care ability *(*β = −*1.468, P*<*0.001)* and social support was a positive predictor of humanistic care ability *(*β = *0.551, P*<*0.001)*. The effect of the path “perceived stress → social support → humanistic care ability” was –0.071 and the path “perceived stress → life satisfaction → social support → humanistic care ability” was –0.129. The results were shown in Table 3 and Figure 2.

**Table 3:** The chain mediating effect of life satisfaction and social support between perceived stress and humanistic care ability.

	**Effect**	***SE*/Boot *SE***	***t***	***P***	**LLCI**	**ULCI**
Total effect	−1.630	0.151^a^	−10.811	< 0.001	−1.926	−1.334
Direct effect	−1.468	0.172^a^	−8.542	< 0.001	−1.805	−1.131
Indirect effect						
Ind 1	0.038	0.103^b^	—	—	−0.154	0.250
Ind 2	−0.129	0.039^b^	—	—	−0.209	−0.057
Ind 3	−0.071	0.029^b^	—	—	−0.141	−0.025

The variables in model 6 are as follow: X = perceived stress, Y = humanistic care ability, M_1_ = life satisfaction, M_2_ = social support; Ind 1: perceived stress → life satisfaction → humanistic care ability; Ind 2: perceived stress → life satisfaction → social support → humanistic care ability; Ind 3: perceived stress → social support → humanistic care ability.

^a^*SE*.

^b^Boot *SE*.

## Discussion

Along with the development of the innovative medical model of “patient-centered” care, an emerging emphasis have laid on improving humanistic care abilities among healthcare workers. They are required to incorporate the element of valuing human beings and humanism in their daily “patient-centered” care. Implementing humanistic care was a basic necessity and a global priority to improve overall healthcare quality. Our previous studies (Deng et al., 2019a,b) have developed an evaluation tool for humanistic care abilities among healthcare workers and investigated their current level of humanistic care abilities. Based on the previous investigation, we design this study aiming to examine the potential factors that may influence the level of humanistic care abilities among healthcare workers. First, we probe the correlations of perceived stress, life satisfaction, and social support with humanistic care ability; and second, we identify the moderating or mediating role of life satisfaction and social support in this correlation. Our findings indicate that social support mediated the relationship between perceived stress and humanistic care ability and that perceived stress can influence humanistic care ability through the chain mediating effect of life satisfaction and social support. These results provide evidence for a potential mechanism by which perceived stress may influence humanistic care ability. A more-targeted and -appropriate intervention could be accordingly developed in future training and education.

Healthcare workers with a high level of perceived stress are less capable of providing high-quality humanistic care in clinical practice. As shown in our findings, it is indicated that perceived stress and humanistic care ability among medical workers are negatively correlated *(r* = −0.373^***^). The multivariate logistic regression analysis also reveals that perceived stress is a risk factor *(B* = −*0.069, B* = −*0.122)* for humanistic care ability. The high level of perceived stress could come from poor working conditions and increasing workload, which hinder the implementation of humanistic care to some extent (Lai et al., 2022). These results are consistent with Ma's et al. (2022) previous studies which show that the humanistic care ability of medical staff is positively correlated with the scores of emotional intelligence including self-emotional assessment and expression, self-emotional management, self-emotional utilization, and others' emotional cognition. Individuals with higher-level abilities of emotional regulation and emotional intelligence are regarded to be more capable of dealing with perceived stress from work and life (Xu et al., 2021). Moreover, in a qualitative study of exploring nurses' perception and their coping strategies with adversity during COVID-19, the humanistic care is reported as a promoting factor of psychological resilience to deal with perceived stress (Jiang et al., 2022). Considered together, the strategies for coping with perceived stress and emotional regulation should be recommended in the training plan to improve humanistic care abilities among healthcare workers.

The other factors including life satisfaction and social support and their interactive relationship should also be carefully considered to promote humanistic care. Our study found a negative correlation between perceived stress with life satisfaction *(r* = −*0.438, shown in* *Figure 1**)* and social support *(r* = −*0.282, shown in* *Figure 1**)*. Similarly, previous studies have reported that they found a negative relationship between perceived stress with life satisfaction (Xu et al., 2021) and social support (Kalaitzaki et al., 2021). In a stressful situation like COVID-19, the social support of feeling respected and understood by society, family, and peers could improve resilience (Jiang et al., 2022). This is also supported by our logistic regression analysis showing that social support is a protective factor for humanistic care ability. In addition, consistent with previous research (Bryson and Bogart, 2020; Cao and Zhou, 2021), we found a relationship between social support and life satisfaction *(r* = −0.454^***^). These results suggest a potential moderating or mediating effect that was hypothesized by our team. To validate our hypothesis, we further conduct the moderating and mediating effect analysis. Although no significant moderating effect is found in both life satisfaction and social support (shown in Table 4), a partial mediating effect of social support is indicated in the connection between perceived stress and humanistic care, and the chain of life satisfaction → social support plays a chain mediating effect. Furthermore, the result suggests that perceived stress could not only be a direct negative predictor of humanistic care ability but also indirectly affect it through life satisfaction and social support (the total mediating effect was −0.200).

**Table 4:** The moderating effect of life satisfaction and social support between perceived stress and humanistic care ability.

**Variables**	**Module 1**				**Module 2**			
	**Coefficient**	***SE***	***P***	**Δ*R*^2^**	**Coefficient**	***SE***	***P***	**Δ*R*^2^**
Constant	153.358	1.096	< 0.00	0.001	153.358	1.096	< 0.0	0.008*
Life satisfaction	—	—	—		0.085	0.188	0.653	
Perceived stress	−1.447	0.170	< 0.001		−1.447	0.170	< 0.001	
Social support	0.511	0.142	< 0.001		—	—	—	
int_1	−0.024	0.200	0.228		—	—	—	
int_2	—	—	—		−0.064	0.022	0.004	

The dependent variable of all these modules is humanistic care ability; int_1: perceived stress × social support; int_2: perceived stress × life satisfaction.

*P < 0.05.

The results of our study indicate that the level of perceived stress, life satisfaction, social support, and humanistic care ability differ across different demographic features in healthcare workers. As shown in Table 1, we found a significant difference between different “job type” (*P* = 0.025) and hospital sources (*P* = 0.004). For hospital source, we could see that the humanistic care abilities among healthcare workers in secondary and tertiary hospitals are significantly higher than those in primary hospitals. This suggests that the healthcare professionals working in senior hospitals could perform better humanistic care in clinical practice. The reasons we assume are first due to the better organizational environment and social support in senior hospitals compared to that in primary ones. Second, the healthcare professionals working in senior hospitals are purposely and carefully selected for higher psychological resilience. As illustrated in Liu's et al. (2022) article, these factors give rise to a set of intertwined impacts to promote the realization of humanistic caring. With respect to job type, our results are peculiar, in that, the humanistic care ability of doctors was significantly higher than that of medical technicians, and the results are statistically significant. This phenomenon may be caused by the differing exposure levels of the two types of healthcare professional to patients. Doctors are in direct contact with patients as they need to treat patients suffering from various types of diseases in their clinical work. It is therefore possible that they are better able to understand the patient's requirements and needs while the medical technicians have less access to patients as they carry out more of their work with tools and conduct inspections with instruments.

Our study was not without limitations. First, the nature of the cross-sectional study does not allow causal relationships to be demonstrated between the variables. Therefore, we would suggest that a longitudinal study is carried out in future. Second, as a convenient sampling method was adopted, the majority of our participants were recruited from military hospitals. This may restrict the generalization of the findings to healthcare workers in civil hospitals. Further studies are required among a larger group of participants and that targeted interventions in respect of stress management need to be developed and rolled out in China. Third, our participants show an unbalanced distribution of the workforce type as the majority of the participants are nurses. The reason may lie in the convenience sampling method that the nurses are more likely to read the advertisement and respond to the survey. The nurses spend most of the time in clinical settings taking care of the inpatient patients while the doctors and the technicians are at outpatients, operating rooms, and laboratories. The technicians are also busy moving among different departments to collect samples.

## Data availability statement

The raw data supporting the conclusions of this article will be made available by the authors, without undue reservation.

## Ethics statement

The study proposal was approved by the Human Ethics Committee for Scientific Research in the Army Medical Center of PLA, which is also known as the Third Affiliated Hospital to the Army Medical University [Medical Research Review (2019) No. 181]. This study was conducted strictly in accordance with the ethical principles of the Declaration of Helsinki. Written informed consent for participation was not required for this study in accordance with the national legislation and the institutional requirements.

## Author contributions

XX and JD: conception and design. ZW and LX: administrative support, data analysis, and interpretation. ZL, LF, and JF: collection and assembly of data. All authors contributed to the article and approved the submitted version.

## Funding

For project 1 this study receieved funding from “Construction of competency-based admission criteria for clinical teaching of nursing undergraduates” (Number: CQGJ19B123, Source: Higher Education Science research project of Chongqing Higher Education Association). For project 2 this study receieved funding from “Effect of emotion regulation training on job burnout of clinical nursing teachers at military medical university” (Number: 2018XRW09, Source: Humanities and Social Science Foundation general project).

## Conflict of interest

The authors declare that the research was conducted in the absence of any commercial or financial relationships that could be construed as a potential conflict of interest.

## Publisher's note

All claims expressed in this article are solely those of the authors and do not necessarily represent those of their affiliated organizations, or those of the publisher, the editors and the reviewers. Any product that may be evaluated in this article, or claim that may be made by its manufacturer, is not guaranteed or endorsed by the publisher.

## References

[B1] Bryson B. A. Bogart K. R. (2020). Social support, stress, and life satisfaction among adults with rare diseases. Health Psychol. 39, 912–920. 10.1037/hea0000905 32584069

[B2] Cao Q. Zhou Y. (2021). Association between social support and life satisfaction among people with substance use disorder: the mediating role of resilience. J. Ethn. Subst. Abuse 20, 415–427. 10.1080/15332640.2019.1657545 31544654

[B3] Deng J. Lei L. Zhang H. L. Luo Y. (2019a). The current status and the influencing factors of humanistic care ability among a group of medical professionals in Western China. Technol. Health Care. 27, 195–208. 10.3233/THC-181389 30562911 PMC6484270

[B4] Deng J. Zhang H. Fang Y. Wang D. Luo Y. (2019b). Research on the construction of an evaluation index system of medical staff's life care ability based on the concept of “people-oriented”. Chin. J. Med. Ethics 1, 122–126. 10.12026/j.issn.1001-8565.2019.01.29

[B5] Diener E. D. Emmons R. A. Larsen R. J. Griffin S. (1985). The satisfaction with life scale. J. Pers. Assess. 49, 71–75. 16367493 10.1207/s15327752jpa4901_13

[B6] Fisher M. H. Sung C. Kammes R. R. Okyere C. Park J. (2022). Social support as a mediator of stress and life satisfaction for people with intellectual or developmental disabilities during the COVID-19 pandemic. J. Appl. Res. Intellect. Disabil. 35, 243–251. 10.1111/jar.12943 34633129 PMC8646736

[B7] França J. R. da Costa S. F. Lopes M. E. da Nóbrega M. M. de França I. S. (2013). The importance of communication in pediatric oncology palliative care: focus on Humanistic Nursing Theory. Rev. Lat. Am. Enfermagem 21, 780–786. 10.1590/S0104-11692013000300018 23918025

[B8] Gao M. Wang Y. Lei Y. Zhang L. Li L. Wang C.. (2021a). Applying the Carolina care model to improve nurses' humanistic care abilities. Am. J. Transl. Res. 13, 3591–3599. 34017540 PMC8129219

[B9] Gao M. Zhang L. Wang Y. Li L. Wang C. Shen Q.. (2021b). Influence of humanistic care based on Carolina care model for ovarian cancer patients on postoperative recovery and quality of life. Am. J. Transl. Res. 13, 3390–3399. 34017514 PMC8129217

[B10] Gater A. Kitchen H. Heron L. Pollard C. Håkan-Bloch J. Højbjerre L.. (2015). Development of a conceptual model evaluating the humanistic and economic burden of Crohn's disease: implications for patient-reported outcomes measurement and economic evaluation. Expert Rev. Pharmacoecon. Outcomes Res. 15, 643–656. 10.1586/14737167.2015.1045883 25985850

[B11] Jiang J. Liu Y. Han P. Zhang P. Shao H. Peng H.. (2022). Psychological resilience of emergency nurses during COVID-19 epidemic in Shanghai: a qualitative study. Front. Public Health 10:1001615. 10.3389/fpubh.2022.1001615 36187606 PMC9524357

[B12] Kalaitzaki A. Tsouvelas G. Koukouli S. (2021). Social capital, social support and perceived stress in college students: the role of resilience and life satisfaction. Stress Health. 37, 454–465. 10.1002/smi.3008 33206451

[B13] Kusier A. O. Folker A. P. (2021). The satisfaction with life scale: philosophical foundation and practical limitations. Health Care Anal. 29, 21–38. 10.1007/s10728-020-00420-y 33386535

[B14] Lai L. Ding S. Zhong Z. Mao P. Sun N. Zheng F. (2022). Association between positive mental character and humanistic care ability in chinese nursing students in Changsha, China. Front. Psychol. 13:896415. 10.3389/fpsyg.2022.896415 35795450 PMC9251419

[B15] Liu X. Li C. Yan X. Shi B. (2022). Psychological capital has a positive correlation with humanistic care ability among nurses. Front. Psychol. 13:955627. 10.3389/fpsyg.2022.955627 36186317 PMC9524352

[B16] Lu F. Xu Y. Yu Y. Peng L. Wu T. Wang T.. (2019). Moderating effect of mindfulness on the relationships between perceived stress and mental health outcomes among chinese intensive care nurses. Front. Psychiatry 10:260. 10.3389/fpsyt.2019.00260 31057445 PMC6482227

[B17] Ma J. Peng W. Pan J. (2022). Investigation into the correlation between humanistic care ability and emotional intelligence of hospital staff. BMC Health Serv. Res. 22:839. 10.1186/s12913-022-08227-4 35773661 PMC9244559

[B18] Mustika R. Soemantri D. (2020). Unveiling the hurdles in cultivating humanistic physicians in the clinical setting: an exploratory study. Malay. J. Med. Sci. 27, 117–124. 10.21315/mjms2020.27.3.12 32684812 PMC7337956

[B19] O'Conoor. (1993). Paterson and Zderad Paterson and Zderad N O'Connor SAGE 64pp £6.50 0-8039-4489-6. Nurs Stand. 7:50. 10.7748/ns.7.32.50.s53 27665832

[B20] Santini Z. I. Koyanagi A. Tyrovolas S. Mason C. Haro J. M. (2015). The association between social relationships and depression: a systematic review. J. Affect. Disord. 175, 53–65. 10.1016/j.jad.2014.12.049 25594512

[B21] She Z. Li D. Zhang W. Zhou N. Xi J. Ju K. (2021). Three versions of the perceived stress scale: psychometric evaluation in a nationally representative sample of chinese adults during the COVID-19 pandemic. Int. J. Environ. Res. Public Health 18:8312. 10.3390/ijerph18168312 34444061 PMC8391348

[B22] Shiau S. J. Chen C. H. (2008). Reflection and critical thinking of humanistic care in medical education. Kaohsiung J. Med. Sci. 24, 367–372. 10.1016/S1607-551X(08)70134-7 18805752 PMC11917884

[B23] Siaw M. Lee J. Y. (2019). Multidisciplinary collaborative care in the management of patients with uncontrolled diabetes: a systematic review and meta-analysis. Int. J. Clin. Pract. 73:e13288. 10.1111/ijcp.13288 30369012

[B24] Silva T. N. (2013). Paterson and Zderad's humanistic theory: entering the between through being when called upon. Nurs. Sci. Q. 26, 132–135. 10.1177/0894318413477209 23575489

[B25] Tan X. Zhang Y. Shao H. (2019). Healthy China 2030, a breakthrough for improving health. Glob. Health Promot. 26, 96–99. 10.1177/1757975917743533 29297762

[B26] Tomás C. C. Oliveira E. Sousa D. Uba-Chupel M. Furtado G. Rocha C.. (2016). Proceedings of the 3rd IPLeiria's International Health Congress: Leiria, Portugal. 6–7 May 2016. BMC Health Serv. Res. 16(Suppl 3):200. 10.1186/s12913-016-1423-5 27409075 PMC4943498

[B27] Tough H. Siegrist J. Fekete C. (2017). Social relationships, mental health and wellbeing in physical disability: a systematic review. BMC Public Health 17:414. 10.1186/s12889-017-4308-6 28482878 PMC5422915

[B28] Wang Y. Zhang Y. Liu M. Zhou L. Zhang J. Tao H.. (2020). Research on the formation of humanistic care ability in nursing students: a structural equation approach. Nurse Educ. Today 86:104315. 10.1016/j.nedt.2019.104315 31896034

[B29] Watson J. (2007). Watson's theory of human caring and subjective living experiences: carative factors/caritas processes as a disciplinary guide to the professional nursing practice. Texto Contexto Enfermagem 16, 129–135. 10.1590/S0104-07072007000100016

[B30] Wu H. L. Volker D. L. (2012). Humanistic nursing theory: application to hospice and palliative care. J. Adv. Nurs. 68, 471–479. 10.1111/j.1365-2648.2011.05770.x 21771046

[B31] Xu X. Chen L. Yuan Y. Xu M. Tian X. Lu F.. (2021). Perceived stress and life satisfaction among chinese clinical nursing teachers: a moderated mediation model of burnout and emotion regulation. Front. Psychiatry 12:548339. 10.3389/fpsyt.2021.548339 34305659 PMC8295563

[B32] Yang C. Xia M. Han M. Liang Y. (2018). Social support and resilience as mediators between stress and life satisfaction among people with substance use disorder in China. Front. Psychiatry 9:436. 10.3389/fpsyt.2018.00436 30386257 PMC6198788

[B33] Zhang H. Gan L. Li X. Shao X. Zuo L. Gao J.. (2021). The Implementation of patient-centered humanistic care for COVID-19 closely contacted hemodialysis patients under the hospital-based group medical quarantine: a brief research report. Front. Psychol. 12:553234. 10.3389/fpsyg.2021.553234 34690846 PMC8531721

[B34] Zhong X. Liu X. Sheng Y. (2021). The effect of the humanistic care teaching model on nurse patient conflict and nurse turnover intention in a pediatric outpatient department: results of a randomized trial. Transl. Pediatr. 10, 2016–2023. 10.21037/tp-21-214 34584871 PMC8429853

